# The Multicomponent Medicinal Product Hepar Compositum Reduces Hepatic Inflammation and Fibrosis in a Streptozotocin- and High-Fat Diet-Induced Model of Metabolic Dysfunction-Associated Steatotic Liver Disease/Metabolic Dysfunction-Associated Steatohepatitis

**DOI:** 10.3390/biomedicines11123216

**Published:** 2023-12-04

**Authors:** Yvonne Burmeister, Kathrin Weyer, Achim Dörre, Bernd Seilheimer

**Affiliations:** 1 Heel GmbH, 76532 Baden-Baden, Germany; yvonneburmeister@gmx.de (Y.B.); bernd.seilheimer@gmail.com (B.S.); 2 Independent Researcher, 14641 Nauen, Germany; achim.doerre@googlemail.com

**Keywords:** non-alcoholic fatty liver disease (NAFLD), non-alcoholic steatohepatitis (NASH), metabolic dysfunction-associated steatotic liver disease (MASLD), metabolic dysfunction-associated steatohepatitis (MASH), liver, fibrosis, inflammation, Hepar compositum, multicomponent, multitarget

## Abstract

Metabolic dysfunction-associated steatotic liver disease (MASLD)—formerly known as non-alcoholic fatty liver disease (NAFLD)—is the most common chronic liver disease worldwide. Since there is currently no approved pharmacotherapy for MASLD, there is an urgent unmet need for efficacious therapeutics for this disease. Hepar compositum (HC-24) is a multicomponent medicinal product that consists of 24 natural ingredients. It has been shown to have anti-inflammatory properties in an obesity-associated MASLD mouse model, but its potential to reduce MASLD-associated fibrosis had not been explored before this study. Here, we investigated the hepatic anti-inflammatory and anti-fibrotic potential of HC-24 in a streptozotocin (STZ)- and high-fat diet (HFD)-induced model of MASLD. Mice received a single injection of low-dose STZ at 2 days of age, followed by HFD feeding from 4 to 9 weeks of age. Mice were treated every second day with HC-24 or daily with the positive control telmisartan from 6 to 9 weeks of age. A non-diseased control group was included as a healthy reference. An explorative small-scale pilot study demonstrated that HC-24 improved liver histology, resulting in a lower NAFLD activity score and reduced liver fibrosis. A subsequent full study confirmed these effects and showed that HC-24 reduced hepatic inflammation, specifically reducing T helper cell and neutrophil influx, and decreased hepatic fibrosis (with qualitatively reduced collagen type I and type III immunopositivity) in the absence of an effect on body and liver weight, blood glucose or liver steatosis. These results show that HC-24 has hepatoprotective, anti-inflammatory, and anti-fibrotic properties in an STZ- and HFD-induced model of MASLD/MASH, suggesting that this multicomponent medicine has therapeutic potential for MASLD patients.

## 1. Introduction

Metabolic dysfunction-associated steatotic liver disease (MASLD)—formerly known as non-alcoholic fatty liver disease (NAFLD) [1]—is the most common chronic liver disease worldwide, currently estimated to affect ~32% of the world population [2]. This new nomenclature for NAFLD has been implemented to better reflect the metabolic component of the disease and to provide an affirmative, non-stigmatizing name for the disease. The prevalence of this disease has been rising, paralleling the increase in obesity and type 2 diabetes, both of which are major risk factors for MASLD [3,4]. MASLD comprises a spectrum of liver diseases, ranging from the relatively benign accumulation of fat in the liver (simple steatosis) to the more advanced metabolic dysfunction-associated steatohepatitis (MASH; hepatic inflammation in addition to fat accumulation)—formerly known as non-alcoholic steatohepatitis (NASH)—with or without hepatic fibrosis. In MASLD patients, liver fibrosis is a major prognostic predictor of long-term outcomes (both liver-related and overall) [5,6], and reduction in fibrosis is, therefore, a commonly used primary endpoint in clinical trials for the treatment of MASLD/MASH. Given that there is currently no approved pharmacotherapy for MASLD, there is an urgent need for effective therapeutics for this disease.

It is well accepted that the underlying process by which simple steatosis progresses to MASH and fibrosis involves multiple interconnecting molecular pathways [7,8]. Steatosis is considered an early event in MASLD that is relatively benign and requires additional stimuli to progress to MASH. MASH-associated inflammation is driven by additional (intra- and extrahepatic) stresses such as lipotoxicity and oxidative stress and involves various immune cell types such as macrophages, T-cells, and neutrophils [9]. Sustained inflammation and hepatocellular stress can lead to hepatocyte damage and subsequent activation of hepatic stellate cells, the major extracellular matrix (ECM)-producing cell type in MASLD-associated fibrosis [10]. The excessive production of ECM proteins such as type I and type III collagen by activated stellate cells, when not adequately balanced by degradation, results in the net accumulation of ECM in the liver, i.e., fibrosis [10].

Due to the multifactorial nature of the MASLD pathogenesis, treatments for MASLD will most likely need to target multiple aspects of the disease. Hepar compositum (HC-24) is a multicomponent, multitarget medicinal product that consists of 24 natural ingredients. It contains a combination of plant extracts, bioactive metabolites, and animal-derived extracts (full composition shown in Supplemental Table S1). HC-24 has a long history of use as a supportive therapy for liver disorders of various origins [11]. An observational study suggests that HC-24 may improve liver function biomarkers (bilirubin and ALT) in viral hepatitis patients [12]. Several components of HC-24 have properties that would be expected to be beneficial for the treatment of MASLD. *Sylibum marianum* (milk thistle), for instance, which contains silymarin (a flavolignan complex), is described to have antidiabetic and hepatoprotective effects and to reduce inflammation and oxidative stress in vivo (reviewed in [13,14]). Similarly, *Taraxacum officinale* (common dandelion) is considered to have antidiabetic properties [15] and has been reported to protect against chemically induced liver toxicity in rats [16]. *Avena sativa* (oat) has also been described to have anti-inflammatory properties [17] and can protect against LPS-induced liver injury [18].

HC-24 has previously been shown to reduce hepatic inflammation in a diet-induced model of obesity-associated MASH (high-fat diet-fed Ldlr-/-.Leiden mice; [19,20]). However, its potential to reduce the development of liver fibrosis remained unclear. Here, we aimed to investigate the effects of HC-24 on hepatic inflammation and fibrosis. For this, we used the STAM mouse model of MASH [21], in which neonatal mice are exposed to low-dose streptozotocin (STZ) followed by high-fat diet feeding from 4 to 9 weeks of age to induce a diabetic phenotype with liver steatosis, inflammation, and fibrosis. We started with a small-scale pilot study to explore the potential of HC-24 to reduce liver disease, followed by a full study to investigate its putative effects on hepatic inflammation and fibrosis in more detail. The angiotensin II receptor blocker telmisartan, which has previously been shown to have anti-steatotic, anti-inflammatory, and anti-fibrotic effects in this model [22], was included as a positive control. In addition to blocking the renin-angiotensin system, telmisartan has also been described to have PPAR-γ and PPAR-δ agonistic activity, all of which are thought to be beneficial for the treatment of MASLD/MASH [23,24].

## 2. Materials and Methods

### 2.1. Animals

C57BL/6 mice (15-day-pregnant females) were obtained from Charles River Laboratories Japan (Yokohama, Kanagawa, Japan) for the pilot study or from Japan SLC Inc. (Shizuoka, Japan) for the main study. Male offspring from these dams were used in this study. All animals in this study were housed and cared for in accordance with the Japanese Pharmacological Society Guidelines for Animal Use. The study protocols were approved by SMC IACUC with approval numbers RP-138 for MNP048-1208-8 (pilot) and RP-138-1 for SMC009-1410-3 (main study). Animals were maintained in a specific pathogen-free facility at a relative humidity of 45 ± 10%, a temperature of 23 ± 2 °C, and a 12 h artificial light and dark cycle (8 am to 8 pm). The animal room was kept at a high pressure (20 ± 4 Pa) to prevent potential contamination of the facility. Mice were group-housed in polymethylpentene cages CL-0133 (CLEA Japan, Shizuoka, Japan) with a maximum of 4 mice per cage and with *ad libitum* access to food and water.

### 2.2. Study Medication

The HC-24 injection solution and the vehicle control were manufactured and bottled in 2.2 mL and 1.1 mL glass ampoules, respectively, by Heel GmbH (Baden-Baden, Germany) according to the international Good Manufacturing Practice (GMP) standards. The ingredients of HC-24 are listed in Supplemental Table S1. Each ampoule of the vehicle control contained 0.9% sodium chloride for injection. The study medication was packaged, shipped, and labeled by Heel GmbH, Germany.

### 2.3. Experimental Design

**Figure 1:**
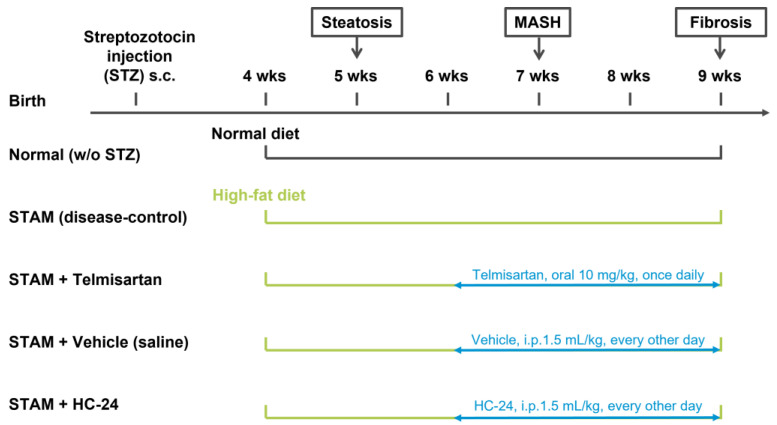
Schematic overview of the experimental design. STAM mice received a subcutaneous (s.c.) injection of streptozotocin (STZ) at 2 days of age and were fed a high-fat diet from 4 weeks of age to induce liver inflammation and fibrosis. From 6 weeks of age, mice in the telmisartan group were treated with telmisartan (once daily oral gavage), mice in the vehicle control group were treated with saline (intraperitoneal (i.p.) injection every other day), and mice in the HC-24 group were treated with HC-24 (i.p. injection every other day). A normal control group (no STZ, standard chow diet) was included as a healthy reference. All mice were terminated at 9 weeks (wks) of age for analyses. This experimental design was used for both the pilot study (*n* = 6 per group) and the main study (*n* = 10 per group).

For the pilot study, 30 male mice were randomized into 5 experimental groups (*n* = 6/group). A non-diseased control group (no STZ treatment, standard chow diet, further referred to as “normal”) was included as a healthy reference. For all other groups, disease was induced by a single subcutaneous injection of streptozotocin at 2 days of age (STZ; Sigma-Aldrich, St. Louis, MO, USA; 200 µg/mouse) and high-fat diet (HFD; 57 kcal % fat, cat#: HFD32, CLEA Japan, Inc., Tokyo, Japan; the composition of this diet is provided in Supplemental Table S2) feeding from 4 weeks of age. This study included an untreated disease-control group and a group treated with telmisartan (10 mg/kg in 10 mL/kg volume by oral gavage once daily, from 6 to 9 weeks of age) as a positive control. HC-24 or saline vehicle (Heel GmbH, Baden-Baden, Germany) was administered by intraperitoneal injection (1.5 mL/kg, every other day) from 6 to 9 weeks of age. Viability, clinical signs, and behavior were monitored daily. Body weight was recorded before treatment. Mice were observed for significant clinical signs of toxicity, moribundity, and mortality for approximately 60 min after each administration. All animals were terminated at 9 weeks of age by exsanguination through direct cardiac puncture under ether anesthesia (Wako Pure Chemical Industries Ltd., Osaka, Japan). For the main study, 50 male mice were randomized into 5 experimental groups (*n* = 10/group). This study included the same groups described above for the pilot study, and the study and treatment durations were the same in the main study as in the pilot study. An overview of the experimental design is provided in Figure 1. One mouse from the telmisartan group of the main study was found dead in week 3 of the study and was excluded from the analysis.

### 2.4. Whole Blood and Plasma Biochemistry

Non-fasting blood glucose was measured in whole blood with a hand-held glucometer (LIFE CHECK, EIDIA Co. Ltd., Tokyo, Japan). For plasma isolation, blood was collected in polypropylene tubes with anticoagulant (Novo-Heparin, Mochida Pharmaceutical, Tokyo, Japan) and centrifuged at 1000× *g* for 15 min at 4 °C. The supernatant was collected and stored at −80 °C until use. Plasma ALT and triglyceride levels were measured by FUJI DRI-CHEM 7000 (Fujifilm, Tokyo, Japan).

### 2.5. Liver Biochemistry

Liver lipid extracts were obtained by Folch’s method [25]. In short, liver samples were homogenized in chloroform–methanol (2:1, *v*/*v*) and incubated overnight at room temperature. After washing with chloroform–methanol–water 8:4:3, *v*/*v*/*v*) the extracts were evaporated to dryness and dissolved in isopropanol. Liver triglyceride levels were measured by Triglyceride E-test (Wako Pure Chemical Industries). Intrahepatic free cholesterol levels were analyzed in the normal, vehicle control, and HC-24 groups by high-performance thin-layer chromatography as described previously [19]. Free cholesterol levels were expressed per mg liver protein, which was analyzed in the same homogenates using the Lowry protein assay [26].

### 2.6. Liver Histopathology

Liver tissue was fixed in Bouin’s solution and embedded in paraffin. Sections were stained with Modified Mayer’s hematoxylin (Lillie’s Modification) (Muto Pure Chemicals Co., Ltd., Tokyo, Japan) and eosin solution (Wako Pure Chemical Industries). The NAFLD activity score (NAS) was determined according to the criteria of Kleiner and Brunt [27]. In short, hepatic steatosis was scored as follows: 0, <5% steatosis; 1, 5–33% steatosis; 2, 33–66% steatosis; or 3, >66% steatosis. Hepatic inflammation was scored as follows: 0, no inflammatory foci observed; 1, <2 foci per 200× field; 2, 2–4 foci per 200× field; or 3, >2 foci per 200× field. For analysis of hepatic fibrosis, liver sections were stained with picro-Sirius red solution (Waldeck GmbH & CO.KG, Münster, Germany). For quantitative analysis of fibrosis area, bright field images of Sirius-red-stained sections were captured around the central vein using a digital camera (DFC280; Leica, Wetzlar, Germany) at 200× magnification, and the positive areas in 5 fields/section were measured using ImageJ software (https://imagej.net/ij/, National Institute of Health, Bethesda, MD, USA).

For immunohistochemistry, cryosections were prepared from the left lateral lobe embedded in Tissue-Tek O.C.T. Compound (Sakura Finetek Japan Co., Ltd., Tokyo, Japan), which were fixed in acetone. Endogenous peroxidase activity was blocked using 0.03% H_2_O_2_ for 5 min, followed by blocking using the blocking reagent Block Ace for 10 min (Dainippon Sumitomo Pharma, Osaka, Japan). Sections were incubated with primary antibodies against F4/80 (1:200; BMA Biomedicals, Augst, Switzerland), CD4 (1:50; Abcam, Waltham, MA, USA), GR-1 (1:50; BD Biosciences, Franklin Lakes, NJ, USA), α-SMA (1:200; Abcam), collagen type 1 (1:2000; LSL, Tokyo, Japan) or collagen type 3 (1:2000, LSL) for 1 h at room temperature. After incubation with a secondary antibody (HRP-Goat anti-rat antibody, Invitrogen, Waltham, MA, USA; or HRP-Goat anti-rabbit antibody, Jackson ImmunoResearch Laboratories, West Grove, PA, USA), enzyme–substrate reactions were performed using DAB peroxidase substrate kit (Nichirei, Tokyo, Japan). Immunohistological stainings were assessed by a board-certified pathologist in a blinded analysis. The number of F4/80-, CD4- and GR-1-positive cells was quantified by counting the number of positive cells in 5 non-overlapping fields (400× magnification for F4/80, 200× magnification for CD4 and GR-1) and expressed as number of positive cells per field. α-SMA was assessed using a semi-quantitative score for portal areas and sinusoids in which the following scores were defined: 0 = no staining, 1 = minimal staining, 2 = mild staining, and 3 = moderated staining. The score for the portal area and the sinusoidal area were added together to provide a total α-SMA staining score.

### 2.7. Statistical Analysis

Statistical analyses were performed in R (version 4.0.2; [28]). In the HC-24 group of the GR-1 staining, 2 mice had extremely high GR-1 values. Based on the GR-1 values of the remaining 8 mice and normal approximation (*p* = 0.395 based on Shapiro–Wilk test), we confirmed these 2 values to be outliers as both were above the 0.1% quantile. Consequently, these 2 samples were excluded from the analysis of GR-1. Data on body weight, Sirius-red-positive area, and F4/80, CD4, GR-1, and α-SMA stainings were tested for normality using the Shapiro–Wilk test. The normality assumption was not rejected for any of the variables (at the 5% significance level) except for the CD4 staining. As normality was rejected for this staining, pairwise comparisons between groups were performed using two-sided Wilcoxon rank sum tests with continuity correction. One-way ANOVA was used to compare the body weights on the final day of the study across all considered groups; the corresponding *p*-value is based on an F-test. For remaining analyses, pairwise comparisons between groups were performed using two-sided Welch’s two-sample *t*-tests with the exception of the NAS, NAS subscores, and the α-SMA score, for which group comparisons were tested using a two-sided Wilcoxon rank sum test with continuity correction in order to account for the ordinal scale of those variables. Statistical significance of differences for pairwise comparisons of all groups is shown in the figures. For brevity, only the comparisons between normal vs. disease-control, disease-control vs. telmisartan, and vehicle control vs. HC-24 are discussed in the text. N.B. The vehicle control did not differ statistically significantly from the disease-control group for any of the parameters reported except for the parameter “plasma triglycerides” of the full study, where *p* = 0.0262. A *p*-value < 0.05 was considered statistically significant. All descriptive summaries are shown as mean ± standard deviation with the exception of the NAS and its components (steatosis score and inflammation score) and the α-SMA score, which are summarized as median and interquartile range (IQR).

## 3. Results

### 3.1. HC-24 Does Not Affect Metabolic Risk Factors but Does Reduce NAFLD Activity Score (NAS) and Fibrosis in a Small-Scale Pilot Study

We first explored the potential of HC-24 to improve liver health in a small-scale pilot study (*n* = 6/group) in STZ and HFD-treated mice. Disease induction with STZ and HFD resulted in a significant reduction in body weight, a significant increase in blood glucose and plasma triglycerides, and a tendency towards an increase in plasma ALT (*p* = 0.06) relative to normal controls (Table 1). Treatment with the positive control telmisartan further lowered body weight (relative to disease-control) and lowered plasma ALT without affecting plasma triglycerides and blood glucose (Table 1). HC-24 treatment did not affect body weight, blood glucose, or plasma ALT and triglycerides relative to vehicle control (Table 1).

**Figure 2:**
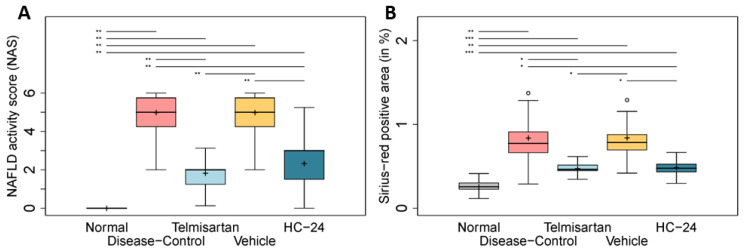
Effects of HC-24 on NAFLD activity score (NAS) and hepatic fibrosis in a small-scale pilot study. Mice (*n* = 6/group) were treated with STZ at 2 days of age and fed a high-fat diet from 4 weeks of age (disease-control). A normal control group (no STZ, standard chow diet) was included as a healthy reference. Telmisartan (treatment from 6 to 9 weeks of age) was included as a positive control. Mice were treated with HC-24 or vehicle control from 6 to 9 weeks of age. (**A**) Histopathology assessed by NAS and (**B**) fibrosis assessment by automated quantification of Sirius-red-stained sections. Data are presented as box–whisker plots (middle line: median; box: 25th and 75th percentiles; cross: mean value; whiskers: first and third quartile −/+ 1.5 × IQR, respectively, or truncated to smallest and largest possible value, where exceeded; white circles: values that are lower than Q1 − 1.5 × IQR or higher than Q1 + 1.5 × IQR). * *p* < 0.05; ** *p* < 0.01; *** *p* < 0.001.

Next, we performed a histopathological analysis of the livers from this pilot study, assessing the effect of HC-24 on the NAS (Figure 2A) and hepatic fibrosis (Figure 2B). Disease induction with STZ and HFD resulted in a significant increase in the NAS (median 0.0, IQR 0.0 in normal; median 5.0, IQR 2.0 in diseased; *p* = 0.003), and treatment with the positive control telmisartan significantly reduced this induction (median 2.0, IQR 1.3 in telmisartan; *p* = 0.005 vs. disease-control). HC-24 treatment significantly lowered the NAS relative to the vehicle control (median 5.0, IQR 2.0 in vehicle; median 3.0, IQR 2.0 in HC-24; *p* = 0.004). Hepatic fibrosis was also significantly induced in the disease-controls (0.3 ± 0.1% in normal; 0.8 ± 0.3% in diseased; *p* = 0.004), and fibrosis area was significantly lowered by telmisartan (0.5 ± 0.1% in telmisartan; *p* = 0.030 vs. disease-control). Treatment with HC-24 significantly lowered hepatic fibrosis relative to vehicle control (0.8 ± 0.2% in vehicle; 0.5 ± 0.1% in HC-24; *p* = 0.014). Based on the results from this pilot study, we continued with a full study to investigate putative effects on hepatic inflammation and fibrosis in more detail using a larger group size (*n* = 10/group).

### 3.2. Effect of Treatments on Metabolic Risk Factors

An overview of the metabolic risk factors body weight, blood glucose, plasma ALT, and plasma triglycerides from this full study is provided in (Table 2). Disease induction with STZ and HFD led to a significant reduction in body weight relative to the normal controls. Blood glucose, plasma ALT, and plasma triglycerides were all significantly increased in the disease-control group relative to normal. Treatment with telmisartan further reduced body weight relative to the disease-controls and did not affect blood glucose, plasma ALT, or triglycerides. In line with the results from the pilot study, HC-24 did not affect body weight, blood glucose, and plasma ALT. Plasma triglycerides were significantly lowered by treatment with HC-24 in comparison with the vehicle.

### 3.3. HC-24 Reduces NAFLD Activity Score (NAS)

**Figure 3:**
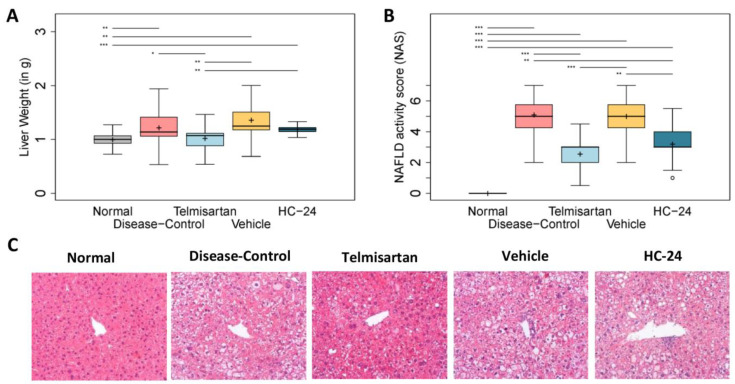
Effects of HC-24 on liver weight and NAS. Mice (*n* = 10/group, except telmisartan-treated group *n* = 9) were treated with STZ at 2 days of age and fed a high-fat diet from 4 weeks of age (disease-control). A normal control group (no STZ, standard chow diet) was included as a healthy reference. Telmisartan (treatment from 6 to 9 weeks of age) was included as a positive control. Mice were treated with HC-24 or vehicle control from 6 to 9 weeks of age. (**A**) Liver weight and (**B**) histopathology assessed by NAFLD activity score (NAS) and (**C**) representative images of hematoxylin and eosin-stained liver sections were assessed at 9 weeks of age (200× magnification). Data are presented as box–whisker plots (middle line: median; box: 25th and 75th percentiles; cross: mean value; whiskers: first and third quartile −/+ 1.5 × IQR, respectively, or truncated to smallest and largest possible value, where exceeded; white circles: values that are lower than Q1 − 1.5 × IQR or higher than Q1 + 1.5 × IQR). * *p* < 0.05; ** *p* < 0.01; *** *p* < 0.001.

Next, we analyzed the effects of HC-24 on liver weight (Figure 3A) and liver histopathology using the NAS (Figure 3B and representative photomicrographs in Figure 3C). Disease induction with STZ and HFD resulted in a significant increase in liver weight relative to the normal controls (1.0 ± 0.1 g in normal; 1.2 ± 0.2 g in diseased; *p* = 0.009), and telmisartan treatment significantly lowered liver weight relative to the disease-controls (1.0 ± 0.1 g in telmisartan; *p* = 0.026 vs. disease-control). Treatment with HC-24 did not reduce liver weight relative to the vehicle control (1.4 ± 0.3 g in vehicle; 1.2 ± 0.1 g in HC-24; *p* = 0.103). As expected, the NAS was increased in the diseased controls relative to normal (median 0.0, IQR 0.0 in normal; median 5.0, IQR 2.0 in diseased; *p* < 0.001). Telmisartan significantly reduced the NAS relative to disease-controls (median 3.0, IQR 2.0 in telmisartan; *p* < 0.001 vs. disease-control). The reduction in the NAS by HC-24 observed in the pilot study was confirmed in the full study, with a significant reduction relative to vehicle in the HC-24-treated animals (median 5.0, IQR 2.0 in vehicle; median 3.0, IQR 1.3 in HC-24; *p* = 0.002). To further specify the effects of HC-24, we next investigated two individual components of the NAS: steatosis and inflammation.

### 3.4. HC-24 Does Not Affect Hepatic Steatosis

**Figure 4:**
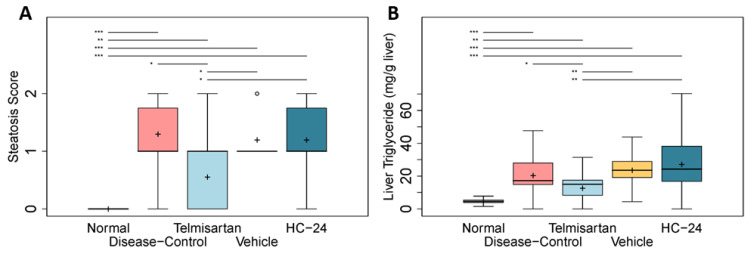
Effects of HC-24 on hepatic steatosis. Mice (*n* = 10/group, except telmisartan-treated group *n* = 9) were treated with STZ at 2 days of age and fed a high-fat diet from 4 weeks of age (disease-control). A normal control group (no STZ and standard chow diet) was included as a healthy reference. Telmisartan (treatment from 6 to 9 weeks of age) was included as a positive control. Mice were treated with HC-24 or vehicle control from 6 to 9 weeks of age. (**A**) Histologically scored liver steatosis and (**B**) biochemically determined hepatic triglycerides were assessed at 9 weeks of age. Data are presented as box–whisker plots (middle line: median; box: 25th and 75th percentiles; cross: mean value; whiskers: first and third quartile −/+ 1.5 × IQR, respectively, or truncated to smallest and largest possible value, where exceeded; white circles: values that are lower than Q1 − 1.5 × IQR or higher than Q1 + 1.5 × IQR). * *p* < 0.05; ** *p* < 0.01; *** *p* < 0.001.

The effects of HC-24 on hepatic steatosis were assessed histologically (by determining steatosis score; Figure 4A) and biochemically (measurement of hepatic triglycerides; Figure 4B). Disease induction resulted in a steatosis score of 1.0 (median, with IQR: 0.75) in disease-controls, which was a significant induction relative to normal (median: 0.0, IQR: 0.0; *p* < 0.001). Telmisartan significantly reduced the presence of steatosis in the liver (median: 1.0, IQR: 1.0 in telmisartan; *p* = 0.010 vs. disease-control). The steatosis score was not affected by HC-24 treatment (median: 1.0, IQR: 0.0 in vehicle; median: 1.0, IQR: 0.75 in HC-24; *p* = 0.962). In line with this, hepatic triglyceride levels were significantly increased in disease-controls relative to normal (4.7 ± 1.0 mg/g tissue in normal; 20.4 ± 8.1 mg/g tissue in diseased; *p* < 0.001), and telmisartan significantly reduced hepatic triglycerides relative to disease-control (12.7 ± 6.9 mg/g tissue in telmisartan; *p* = 0.040 vs. disease-control). HC-24 did not affect triglyceride accumulation in the liver (23.6 ± 6.6 in vehicle; 27.2 ± 11.7 in HC-24; *p* = 0.412).

### 3.5. HC-24 Reduces Hepatic Inflammation

**Figure 5:**
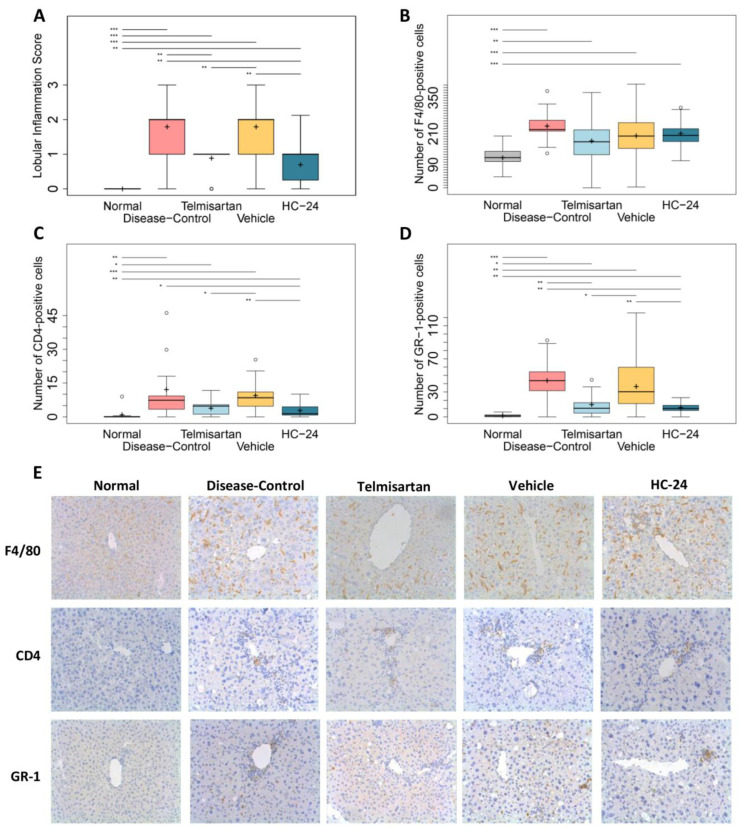
Effects of HC-24 on hepatic inflammation. Mice (*n* = 10/group, except telmisartan-treated group *n* = 9) were treated with STZ at 2 days of age and fed a high-fat diet from 4 weeks of age (disease-control). A normal control group (no STZ, standard chow diet) was included as a healthy reference. Telmisartan (treatment from 6 to 9 weeks of age) was included as a positive control. Mice were treated with HC-24 or vehicle control from 6 to 9 weeks of age. (**A**) Histologically scored liver inflammation and quantification of (**B**) the number of F4/80-positive cells (macrophages) per 400× field, (**C**) the number of CD4-positive cells (T-cells) per 200× field, (**D**) the number of GR-1-positive cells (neutrophils) per 200× field and (**E**) representative images of these immunohistochemical stainings assessed at 9 weeks of age (all 200× magnification). Data are presented as box–whisker plots (middle line: median; box: 25th and 75th percentiles; cross: mean value; whiskers: first and third quartile −/+ 1.5 × IQR, respectively, or truncated to smallest and largest possible value, where exceeded; white circles: values that are lower than Q1 − 1.5 × IQR or higher than Q1 + 1.5 × IQR). * *p* < 0.05; ** *p* < 0.01; *** *p* < 0.001.

Next, we analyzed the effects of HC-24 on hepatic inflammation by analysis of the NAS component score for inflammation (Figure 5A) and by immunohistological analysis of specific immune cell subtypes (Figure 5B–E). The combined STZ and HFD treatment resulted in a significant increase in the inflammation score in disease-controls relative to normal (median: 0.0, IQR: 0.0 in normal; median: 2.0, IQR: 1.0 in diseased; *p* < 0.001) and the inflammation score was significantly reduced in the telmisartan group (median: 1.0, IQR: 0.0 in telmisartan; *p* = 0.007 vs. disease-control). Treatment with HC-24 also significantly reduced hepatic inflammation (median: 2.0, IQR: 1.0 in vehicle; median: 1.0, IQR: 0.75 in HC-24; *p* = 0.003). To further characterize the observed anti-inflammatory effect of HC-24, we investigated its effect on macrophages (F4/80), T-cells (CD4), and neutrophils (GR-1) by an immunohistological analysis. This analysis showed that the number of F4/80-positive cells (Figure 5B) was significantly increased in disease-controls relative to normal (117 ± 26 in normal; 241 ± 67 in disease-control; *p* < 0.001). Neither telmisartan (183 ± 55 in telmisartan; *p* = 0.061 vs. disease-control) nor HC-24 (203 ± 56 in vehicle control; 211 ± 44 in HC-24; *p* = 0.695) affected the presence of F4/80-positive cells. The number of CD4-positive T-cells (Figure 5C) was significantly increased in the liver lobule of disease-controls relative to normal (1.0 ± 2.8 in normal; 12.2 ± 14.4 in disease-control, *p* = 0.001), and their presence was not significantly affected by telmisartan (3.9 ± 2.7 in telmisartan, *p* = 0.055 vs. disease-control). HC-24 treatment significantly lowered the number of CD4-positive cells relative to vehicle controls (9.5 ± 7.0 in vehicle control; 2.9 ± 3.1 in HC-24; *p* = 0.005). Disease induction in the disease-controls was accompanied by a significant increase in the number of GR-1-positive cells (Figure 5D) relative to normal controls (1.5 ± 1.7 in normal; 44 ± 23 in disease-controls, *p* < 0.001). The number of GR-1-positive cells was significantly reduced in telmisartan-treated animals (15.1 ± 14.1 in telmisartan; *p* = 0.004 vs. disease-control) and in HC-24-treated animals (36.6 ± 23.8 in vehicle control; 11.4 ± 6.1 in HC-24; *p* = 0.009) relative to their respective controls.

Since HC-24 has previously been described to have anti-inflammatory effects that were associated with a reduction in the intrahepatic accumulation of free cholesterol [19], we next investigated the effects of HC-24 on hepatic free cholesterol as a potential underlying mechanism for the observed anti-inflammatory effects in this study. Biochemical analysis of intrahepatic free cholesterol showed that free cholesterol levels were 8.3 ± 2.0 µg/mg liver protein in normal, and vehicle controls had comparable levels (8.6 ± 1.0 µg/mg liver protein; *p* = 0.91). HC-24 treatment did not affect the accumulation of hepatic free cholesterol (8.5 ± 1.2 µg/mg liver protein; *p* = 0.99 vs. vehicle control).

### 3.6. HC-24 Reduces Hepatic Fibrosis

**Figure 6:**
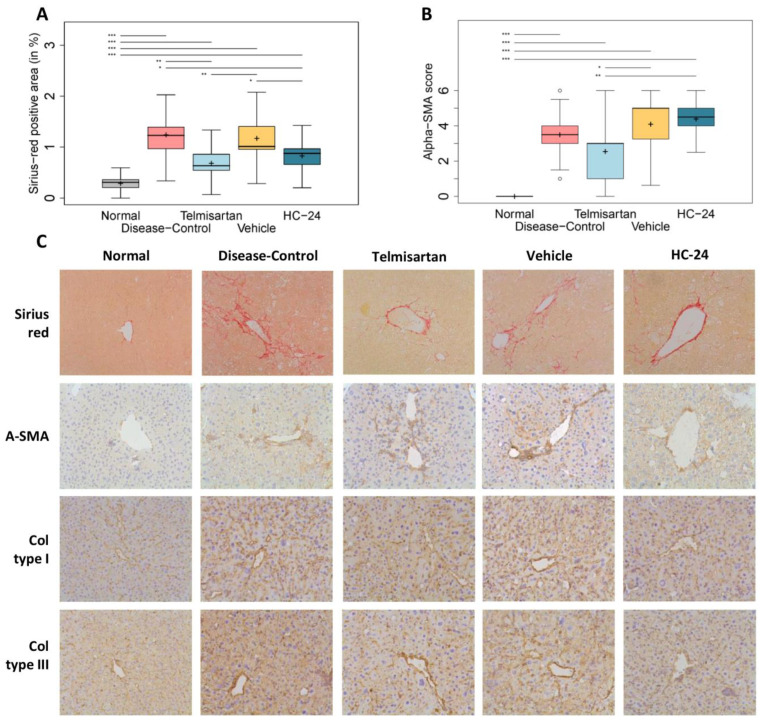
Effects of HC-24 on hepatic fibrosis. Mice (*n* = 10/group, except telmisartan-treated group *n* = 9) were treated with STZ at 2 days of age and fed a high-fat diet from 4 weeks of age (disease-control). A normal control group (no STZ and standard chow diet) was included as a healthy reference. Telmisartan (treatment from 6 to 9 weeks of age) was included as a positive control. Mice were treated with HC-24 or vehicle control from 6 to 9 weeks of age. (**A**) Automated quantification of Sirius-red-stained liver sections, (**B**) pathologist-assessed semi-quantification of α-SMA-stained liver sections, and (**C**) representative images of Sirius-red-stained sections and immunohistochemical stainings for hepatic stellate cell activation marker α-SMA, type I collagen and type III collagen assessed at 9 weeks of age (all 200× magnification). Data are presented as box–whisker plots (middle line: median; box: 25th and 75th percentiles; cross: mean value; whiskers: first and third quartile −/+ 1.5 × IQR, respectively, or truncated to smallest and largest possible value, where exceeded; white circles: values that are lower than Q1 − 1.5 × IQR or higher than Q1 + 1.5 × IQR). * *p* < 0.05; ** *p* < 0.01; *** *p* < 0.001.

Finally, we investigated the effects of HC-24 on hepatic fibrosis by quantitative and semi-quantitative analysis of Sirius-red-stained liver sections and hepatic stellate cell activation, respectively (Figure 6A,B with representative photomicrographs in Figure 6C) and qualitative immunohistological analysis of collagens (Figure 6C). Image analysis of Sirius-red-stained sections showed that, as expected, disease induction resulted in a significant increase in Sirius-red-positive area (0.3 ± 0.1% in normal; 1.2 ± 0.4% in diseased; *p* < 0.001). The Sirius-red-positive area was significantly reduced by telmisartan treatment (0.7 ± 0.2% in telmisartan; *p* = 0.002 vs. disease-control). HC-24 treatment reduced the Sirius-red-positive area relative to the vehicle (1.2 ± 0.4% in vehicle; 0.8 ± 0.2% in HC-24; *p* = 0.027), thereby confirming the anti-fibrotic effect of HC-24 observed in the pilot study. Semi-quantitative analysis of α-SMA immunoreactivity as a marker of hepatic stellate cell activation (Figure 6B) revealed a significant increase in α-SMA-positive staining in the disease-controls relative to normal (median: 0.0, IQR: 0.0 in normal; median: 3.5, IQR 1.0 in disease-control; *p* < 0.001). The degree of α-SMA staining was not affected by telmisartan (median 3.0, IQR 2.0 in telmisartan; *p* = 0.180 vs. disease-control) or HC-24 (median 5, IQR 1.75 in vehicle control; median 4.5, IQR 1.0 in HC-24; *p* = 0.968). Immunohistological staining for collagen type I and type III showed an apparent increase in sinusoidal deposition of both collagens in disease-control and vehicle relative to normal. Telmisartan and HC-24 both qualitatively reduced the presence of these collagen types relative to their respective controls, thus confirming their anti-fibrotic effect.

## 4. Discussion

This study shows that treatment with the multicomponent medicinal product HC-24 has multitarget anti-inflammatory and anti-fibrotic effects in an STZ- and HFD-induced model of MASLD/MASH. We could show that HC-24 treatment results in a reduction in the NAS, reducing inflammatory cell infiltrates in the liver, specifically reducing the presence of neutrophils and T helper cells. This anti-inflammatory effect was associated with a reduction in hepatic fibrosis and was found in the absence of an effect on steatosis.

In line with what was observed previously in a diet-induced model of obesity-associated MASLD/MASH [19], HC-24 did not affect body weight, blood glucose, or hepatic steatosis but did have a clear anti-inflammatory effect within the liver. Similar to what was reported in that previous study, we found that HC-24 specifically reduced the infiltration of neutrophils (GR-1-positive cells) in the liver without affecting F4/80 immunoreactivity, i.e., the number of macrophages. Neutrophils are generally considered to have a detrimental effect on chronic inflammatory diseases through the production of reactive oxygen species (ROS), cytokines and chemokines, proteases, and neutrophil extracellular traps (NETs) [9]. In human MASH, their infiltration is considered a key histological feature of the disease [29,30]. Neutrophil-produced ROS can contribute to tissue damage through reactions with a wide range of biological substrates such as DNA, proteins, and lipids. In addition, neutrophils and ROS have been described to activate hepatic stellate cells and thereby promote the development and progression of liver fibrosis [31,32,33]. In line with this notion, genetic knockout or therapeutic inhibition of myeloperoxidase (MPO), one of the primary ROS-producing enzymes in neutrophils, reduces high-fat and high-cholesterol-induced liver fibrosis in mice [34]. In addition to this attenuating effect on neutrophil infiltration, HC-24 was also found to reduce the hepatic infiltration of T helper cells (Th-cells; CD4-positive cells). These cells are known to be increased in the liver in human MASH [35,36], and the depletion of T helper cells in humanized MASH mice has been shown to reduce cytokine production and fibrosis progression [37]. The attenuating effect of HC-24 on neutrophil and Th-cell infiltration may thus (partly) underlie the observed anti-fibrotic effect of the treatment. Of note, the anti-inflammatory effect of HC-24 on neutrophils appears to be independent of the underlying disease-inducing mechanisms (i.e., it is both observed in the STZ- and HFD-induced STAM model described herein and in the diet-induced Ldlr-/-.Leiden model that was previously described [19]). Evidence that the anti-inflammatory effect of HC-24 is independent of the underlying disease-inducing mechanism is further provided by the lack of an effect on intrahepatic free cholesterol accumulation in this study. In the previous study, increased cholesterol synthesis and the subsequent accumulation of free cholesterol in the liver were found to drive hepatic inflammation, and the observed anti-inflammatory effects of HC-24 were associated with a reduction in the synthesis and accumulation of this lipotoxic lipid species [19]. In the STZ- and HFD-induced model used in this study, we did not observe an accumulation of intrahepatic free cholesterol, indicating that hepatic inflammation results from other pro-inflammatory drivers in this model. Although the mechanisms underlying the inflammatory response in STZ-induced liver injury have not been completely elucidated, it is thought that free radicals generated in the metabolism of STZ lead to DNA and chromosomal damage, which result in hepatocyte cell death and a subsequent inflammatory response [38].

For the first time, we show that in addition to anti-inflammatory properties, HC-24 also attenuates the development of fibrosis. This is an important finding since fibrosis is a key prognostic marker of mortality and liver-related morbidity in MASLD, and the risk of severe liver disease and mortality increases per stage of fibrosis [5,6,39,40]. We found that HC-24 reduced the total fibrotic area as assessed by Sirius-red staining, and immunohistological analyses point to a specific reduction in the fibrillar collagens type I and type III, both of which are known to be increased in human liver fibrosis [41].

MASH pathophysiology is complex, involving multiple intrahepatic and extrahepatic factors that may act in a parallel or sequential fashion and that can differ within the spectrum of disease or change dynamically over time [42]. Therefore, the prevailing opinion is that combination therapy will be key for the treatment of MASH [43,44]. A single-target approach is not expected to be sufficient for the treatment of MASH, a notion that is further supported by reports on several (single-target) molecules with different mechanisms of action that have been shown to have limited clinical efficacy to date. To improve the results of pharmacological intervention, the treatment of MASH is expected to require the engagement of several targets [43]. The combination of agents, such as in the multicomponent, multitarget medicinal product HC-24, therefore, seems to be a logical approach to the treatment of this complex disease.

A limitation of this study is that the STAM mouse model employed herein is a relatively aggressive model in which disease is induced rapidly. Although it does recapitulate certain histological aspects (e.g., inflammation, fibrosis) of MASLD/MASH, it does not reflect the obese metabolic phenotype observed in patients with MASH [45]. In contrast with what is observed in many MASLD/MASH patients, STAM mice are lean and normolipidemic, and the pancreatic beta-cell toxicity caused by STZ results in a type 1 diabetic phenotype (in which the pancreas is unable to produce insulin) rather than a type 2 diabetic phenotype (in which tissues become insulin resistant). However, the findings from this study do add to the evidence for the hepatoprotective effects of HC-24 and confirm the anti-inflammatory effects observed in a translational, diet-induced model of MASLD/MASH [19]. In addition to its anti-inflammatory effect and despite the harsh nature of the STAM model, HC-24 was able to reduce the development of fibrosis in these mice, providing evidence for its hepatoprotective properties even under these aggressive disease conditions.

Overall, we show that HC-24 reduces hepatic inflammation, limiting the influx of T helper cells and neutrophils in the liver, and reduces hepatic fibrosis in an STZ- and HFD-induced model of MASLD/MASH. These findings provide further support for the use of HC-24 as a treatment for inflammation and fibrosis in MASLD patients.

## 5. Patents

A patent application in relation to results has been filed as International Application No. PCT/EP2021/0730706 (published as WO2022/043479), resulting in national patent applications EP4204085, NC2023/0002354, and RU2023106840.

## Figures and Tables

**Table 1:** Effects of HC-24 on body weight and metabolic risk factors in a small-scale pilot study.

	Normal	Disease-Control	Telmisartan	Vehicle	HC-24
Body weight (g)	25.0 ± 1.6	21.4 ± 1.6 **	18.2 ± 1.8 ^##^	21.2 ± 2.4	20.8 ± 1.4
Whole blood glucose (mg/dL)	162.8 ± 20.8	572.8 ± 118.5 **	659.2 ± 236.7	585.8 ± 72.6	560.3 ± 108.2
Plasma ALT (U/L)	36.2 ± 17.0	62.3 ± 22.4	42.5 ± 5.9 ^#^	62.2 ± 24.2	39.5 ± 13.8
Plasma triglycerides (mg/dL)	94.8 ± 34.4	587.0 ± 439.6 **	937.5 ± 664.6	838.3 ± 607.7	447.0 ± 365.9

Data shown are mean ± SD at the end of the study. * Indicates significance for the comparison disease-control vs. normal (** *p* < 0.01), ^#^ indicates significance for the comparison telmisartan vs. disease-control (^#^ *p* < 0.05; ^##^ *p* < 0.01), and † indicates significance for the comparison HC-24 vs. vehicle.

**Table 2:** Effects of HC-24 on body weight and metabolic risk factors.

	Normal	Disease-Control	Telmisartan	Vehicle	HC-24
Body weight (g)	22.4 ± 1.2	18.9 ± 1.8 ***	16.3 ± 0.7 ^##^	18.6 ± 1.3	18.3 ± 0.9
Whole blood glucose (mg/dL)	168.4 ± 28.3	560.2 ± 121.8 ***	659.8 ± 177.7	556.9 ± 109.8	556.5 ± 112.3
Plasma ALT (U/L)	20.9 ± 7.8	71.3 ± 59.1 *	46.9 ± 14.7	158.9 ± 167.2	87.1 ± 49.6
Plasma triglycerides (mg/dL)	75.9 ± 24.7	225.8 ± 186.2 *	360.6 ± 394.8	550.0 ± 365.7	266.0 ± 122.0 ^†^

Data shown are mean ± SD at the end of the study. * Indicates significance for the comparison disease-control vs. normal (* *p* < 0.05; *** *p* < 0.01), ^#^ indicates significance for the comparison telmisartan vs. disease-control (^##^ *p* < 0.01), and ^†^ indicates significance for the comparison HC-24 vs. vehicle (^†^ *p* < 0.05).

## Data Availability

The data presented in this study are available within this article and in the associated Supplemental Material.

## Supplementary Materials

The following supporting information can be downloaded at https://www.mdpi.com/article/10.3390/biomedicines11123216/s1, Table S1: Composition of HC-24; Table S2: high-fat diet composition.
