# Supplementary material for: The Multicomponent Medicinal Product Hepar Compositum Reduces Hepatic Inflammation and Fibrosis in a Streptozotocin- and High-Fat Diet-Induced Model of Metabolic Dysfunction-Associated Steatotic Liver Disease/Metabolic Dysfunction-Associated Steatohepatitis
Source: Biomedicines. 2023 Dec 4;11(12):3216. doi: 10.3390/biomedicines11123216 (PMC10740479; doi:10.3390/biomedicines11123216)
Supplement: Supplementary file 1 [file biomedicines-11-03216-s001.zip › Supplemental Table S1.pdf]

Supplemental table S1. Composition of HC-24

Component	Manufacturing method (Ph. Eur.)	µg/ml in HC-24
Plant extracts		
Avena sativa L. (Oat), fresh aerial parts	Method 1.1.1	0,02
Chelidonium majus L. (Greater celandine), fresh rhizome and adherent roots	Method 1.1.5	0,3
Cinchona pubescens (Cinchona), dried bark	Method 1.1.8	10
Cynara scolymus L. (Artichoke), fresh epigeal flowering parts	Method 1.1.5	0,03
Lycopodium clavatum L. (Club moss), dried ripe spores	Method 1.1.8	10
Silybum marianum (Milk thistle), mature fruit devoid of the pappus of the fruit	Ph. Eur 8.0/1860	10
Taraxacum officinale (Dandelion), whole fresh flowering plants	Method 1.1.3	2
Veratrum album L (White hellebore), dried rhizome and adherent roots	Method 1.1.8	10
Chemical substances		
α-ketoglutaric acid	Method 3.1.1	1E-6
Fumaric acid	Method 3.1.1	1E-6
Malic acid	Method 3.1.1	1E-6
Orotic Acid Monohydrate	Method 4.1.1	0,01
Thioctic acid	Method 4.1.1	1E-4
Calcium carbonicum Hahnemanni (inner parts of broken shells of the oyster)	Method 4.1.1	1E-24
Cyanocobalamin (vitamin B12)	Method 3.1.1	1
Histamin	Method 3.1.1	1E-6
Diethyloxalacetate, sodium	Method 4.1.1	1E-6
Sulphur	Method 4.1.1	1E-9
Extracts of organs from healthy pigs		
Liver	Method 2.1.1	1E-4
Colon	Method 2.1.1	1E-6
Duodenum	Method 2.1.1	1E-6
Pancreas	Method 2.1.1	1E-6
Thymus	Method 2.1.1	1E-6
Gall bladder	Method 2.1.1	1E-6

Ph. Eur. = European Pharmacopoeia
