# Supplementary material for: The Multicomponent Medicinal Product Hepar Compositum Reduces Hepatic Inflammation and Fibrosis in a Streptozotocin- and High-Fat Diet-Induced Model of Metabolic Dysfunction-Associated Steatotic Liver Disease/Metabolic Dysfunction-Associated Steatohepatitis
Source: Biomedicines. 2023 Dec 4;11(12):3216. doi: 10.3390/biomedicines11123216 (PMC10740479; doi:10.3390/biomedicines11123216)
Supplement: Supplementary file 1 [file biomedicines-11-03216-s001.zip › Supplemental Table S2.pdf]

Supplemental table S2: high-fat diet composition

Formulation (%)	
Milk casein	24.5
Egg white	5.0
L-cystine	0.43
Powdered beef tallow (including 80% of beef tallow)	15.88
Safflower oil (high oleic acid)	20.0
Crystalline cellulose	5.5
Maltodextrin	8.25
Lactose	6.928
Sucrose	6.75
AIN93 vitamin mix	1.4
AIN93G mineral mix	5.0
Choline bitartrate	0.36
Tertiary butylhydroquinone	0.002
